# Pre-Analytical Errors Before, During, and After the COVID-19 Pandemic: A Multi-Year Retrospective Observational Study

**DOI:** 10.3390/healthcare14193286

**Published:** 2026-10-02

**Authors:** Betül Özbek Kurtul, Bağnu Dündar, Fatma Bozkurt, Sevgi Koçyiğit Sevinç, Çiğdem Aktaş, Asiye Gök Yurttaş

**Affiliations:** 1 Department of Medical Biochemistry, Faculty of Medicine, Istanbul Atlas University, Istanbul 34403, Türkiye; betul.ozbek@atlas.edu.tr (B.Ö.K.); bagnu.dundar@atlas.edu.tr (B.D.); 2 Department of Infectious Diseases and Clinical Microbiology, Faculty of Medicine, Istanbul Atlas University, Istanbul 34403, Türkiye; fatma.bozkurt@atlas.edu.tr; 3 Department of Biophysics, Faculty of Medicine, Kutahya Health Sciences University, Kütahya 43100, Türkiye; sevgi.kocyigitsevinc@ksbu.edu.tr; 4 Department of Genetics, Aziz Sancar Institute of Experimental Medicine, Istanbul University, Istanbul 34452, Türkiye; cigdemaktas485@gmail.com

**Keywords:** pre-analytical errors, specimen rejection, COVID-19 pandemic, hemolysis, clotted specimen, laboratory quality indicators, emergency department, intensive care unit

## Abstract

Objective: The pre-analytical phase is the stage at which laboratory errors most frequently occur and is therefore of critical importance for patient safety. During the COVID-19 pandemic, the marked increase in workload, organizational restructuring, staff and resource redeployment, changes in patient volume and clinical workflows, and disease-related pathophysiological alterations affecting specimen characteristics created unprecedented conditions that could influence laboratory quality indicators. This study aimed to characterize changes in specimen rejection rates across the pre-pandemic, pandemic, and normalization periods between 2018 and 2023, and to determine whether pandemic-related disruptions in health care delivery were associated with differences in the number, rate, and distribution of specimen rejections according to clinical unit, rejection reason, and time interval, with emphasis on the magnitude of these differences rather than statistical significance alone. The study also sought to generate evidence that could inform the development of preventive systems and strengthen laboratory preparedness for future large-scale disruptions to health care services. Materials and Methods: This retrospective observational study used routinely collected, aggregated laboratory information system (LIS) data from the Central Laboratory of Istanbul Atlas University Hospital for 1 January 2018 through 31 December 2023. The LIS extract was restricted to eligible specimen types, including spot and 24 h urine, serum, and whole-blood or plasma specimens collected in EDTA-, citrate-, or heparin-containing tubes; all other specimen types were excluded. Specimen rejection rates were evaluated by study period, clinical unit, time interval, and rejection reason. For cross-period comparisons, the total number of specimens within each respective study period was used as the denominator for calculating rejection rates, whereas for rejection-reason-specific analyses, the number of specimen types relevant to each rejection category was used as the denominator. Negative-binomial regression with denominator-based offsets estimated adjusted rejection-rate ratios (RRs) with 95% confidence intervals (CIs), with Bonferroni adjustment for selected pairwise comparisons. No missing or unclassifiable values were identified; no imputation was required. Reporting follows STROBE and the RECORD extension for studies using routinely collected health data. Results: The overall rejection rate increased progressively from 0.23% in the pre-pandemic period to 0.49% during the pandemic and 0.65% in the normalization period, and monthly observed rates supported this progressive increase. In adjusted terms, the rejection rate was 2.20-fold higher during the pandemic than in the pre-pandemic period (95% CI: 1.96–2.46) and increased a further 1.42-fold in the normalization period compared with the pandemic period (95% CI: 1.27–1.59). Across clinical units, the emergency department had the highest observed rejection rate, whereas intensive care units showed the highest adjusted rejection rates during the pandemic (RR = 1.34 versus the emergency department, 95% CI: 1.00–1.80) and normalization periods. Hemolysis and clotting were the dominant rejection reasons; although clotting had the highest observed rates in the pre-pandemic and pandemic periods, hemolysis ranked highest after adjustment in all periods and separated significantly from clotting only in the normalization period (RR = 1.97, 95% CI: 1.65–2.35). Time-interval effects were context-dependent rather than uniform and became informative mainly in combination with clinical unit or rejection reason: intensive care unit rejections peaked in the daytime interval during the pandemic (RR = 1.84 versus the evening interval, 95% CI: 1.06–3.19), and in the normalization period emergency department–hemolysis emerged as the leading adjusted clinical unit–rejection reason combination (RR = 1.89 versus intensive care unit–hemolysis, 95% CI: 1.19–3.00), while clotting remained centered on intensive care units and became more concentrated in the overnight and early-morning interval. Conclusions: Pre-analytical specimen rejection increased progressively across the pre-pandemic, pandemic, and normalization periods, with adjusted effect sizes indicating a substantial and sustained deterioration rather than a transient pandemic-related disturbance, and with persistent differences in rejection patterns according to clinical unit, time interval, and rejection reason. The further increase observed in the normalization period suggests that pandemic-related disruptions were associated with sustained changes in pre-analytical performance but were unlikely to be the sole explanation. Emergency department–hemolysis during the normalization period and intensive care unit–related clotting emerged as the most concrete quality-monitoring targets. Integrating rejection rates and counts with stratified monitoring by clinical unit, time interval, and rejection reason in combination may support earlier identification of emerging risks, more targeted preventive interventions, and stronger laboratory preparedness for future large-scale disruptions to health care services.

## 1. Introduction

Laboratory medicine is a cornerstone of clinical decision-making, and the accuracy of laboratory results is critical to diagnosis, treatment selection, and patient monitoring. Nevertheless, it is well established that the great majority of laboratory errors originate not in the analytical phase itself, but in the pre-analytical phase, which encompasses specimen collection, labeling, transport, storage, and processing prior to analysis [1,2]. As the most fragile link in the total testing process, the pre-analytical phase is particularly vulnerable to error, and mistakes occurring at this stage can directly compromise the reliability of test results, contributing to inappropriate clinical decisions, diagnostic delay, and threats to patient safety.

The specimen rejection rate is widely regarded as one of the most informative quality indicators of the pre-analytical process. Hemolysis, clot formation, insufficient specimen volume, and inappropriate collection conditions are consistently reported among the most frequent causes of specimen rejection [2,3,4]. The frequency of these error types is not uniform but varies considerably according to clinical unit, time of day, specimen volume, and overall workload, with pre-analytical error rates reported to be particularly elevated in high-acuity, high-throughput settings such as the emergency department and the intensive care unit [5,6]. Beyond patient volume, factors such as sample collection technique, staff experience, shift intensity, and broader organizational processes have also been identified as important determinants of how pre-analytical errors are distributed across a health care system.

The COVID-19 pandemic, formally declared by the World Health Organization on 11 March 2020, precipitated far-reaching changes in health care systems worldwide [3]. The surge in patient volume, the redeployment of health care personnel, intensified working conditions, and the implementation of new biosafety requirements during this period directly affected laboratory operations. Because of their high testing volumes and the expectation of rapid turnaround, clinical laboratories were among the units that experienced the greatest operational strain during the pandemic. Importantly, this strain was not limited to operational pressure alone: health care workers during this period have been shown to experience substantial burnout, anxiety, and increased cognitive load [7], and because pre-analytical tasks depend on standardization, sustained attention, and consistent execution of repetitive technical steps, this psychological burden may itself have represented a meaningful, independent threat to pre-analytical quality.

A further, biologically distinct dimension of the pandemic’s potential impact on pre-analytical processes relates to the direct physiological effects of SARS-CoV-2 infection. COVID-19 has been shown to be associated with endothelial injury, microvascular inflammation, hypercoagulability, and thromboinflammatory processes [8,9]. In particular, the vascular disturbances and coagulation activation observed in critically ill patients with COVID-19 may have created a biological substrate capable of impairing specimen quality, independent of any change in staff behavior. For this reason, changes in pre-analytical error patterns observed during the pandemic cannot be attributed to increased workload alone but should instead be considered in light of the combined, multidimensional influence of patient biology and pandemic-related operational conditions.

The extent to which the pandemic affected pre-analytical error rates, and the degree to which any such effect has persisted into the normalization period, remains incompletely understood. A rise in the overall rate of pre-analytical errors and specimen rejection during the acute phase of the pandemic, relative to the pre-pandemic period, has previously been reported [10], yet the majority of the existing literature has focused on overall hemolysis rates or aggregate specimen rejection rates considered in isolation. Long-term studies that jointly examine clinical unit, time interval, and rejection cause—and that evaluate the pre-pandemic, pandemic, and normalization periods within a single, unified analytic framework—remain scarce, underscoring the need for a more detailed investigation of this question.

The objectives, the definitions of the three study periods, the analytical groupings for clinical unit, time interval and rejection reason, and the denominators used for each rate were specified before the data were analyzed and are reported as such; no hypothesis was tested that had not been defined in advance, and no additional grouping was introduced after inspection of the results. In the present study, data from the Central Laboratory of Istanbul Atlas University Hospital were evaluated across three periods: pre-pandemic, pandemic, and normalization. The primary aim of this study was to characterize how specimen rejection rates changed across these periods, and to analyze the relationship between this change and clinical unit, time interval, and rejection reason. In addition, the study aimed to interpret the observed changes in laboratory quality indicators in the context of potential organizational, biological, and psychological dimensions, thereby laying the groundwork for new approaches that could inform laboratory quality management during future periods of health system crisis.

## 2. Material and Methods

### 2.1. Study Design and Setting

This multi-year retrospective observational study was conducted at the Central Laboratory of Istanbul Atlas University Hospital using routinely collected health data extracted from the laboratory information system (LIS). Data were obtained for the period between 1 January 2018 and 31 December 2023. Throughout the study period, the principal laboratory workflows remained largely stable. The laboratory was supervised by the same responsible medical biochemistry specialist from early 2017 through September 2025, providing continuity in oversight of the pre-analytical, analytical, and post-analytical processes. Blood collection was performed using standardized evacuated blood-collection systems across clinical units. Personnel involved in blood collection, including nurses and phlebotomists, received annual training, with additional orientation training for newly recruited staff and targeted retraining when monthly rejection monitoring indicated an increase in rejection counts. Laboratory consumables were procured through the same contracted distributor, and the types and manufacturers of blood-collection tubes remained largely consistent throughout the study period; BD tubes were routinely used for serum-separator, clot-activator serum, and K_2_EDTA whole-blood specimens, whereas Vacusera tubes were used for heparin-, sodium-fluoride-, additive-free, and sodium-citrate specimens, with Vacuette citrate tubes used only occasionally. Specimen-transport procedures also remained stable: most specimens from inpatient wards, intensive care units, and the emergency department were transported using the same pneumatic tube system, while a smaller proportion were delivered directly by specimen-transport personnel. The principal coagulation, hematology, clinical chemistry, and immunoassay platforms likewise remained unchanged during the study period. No major change in these procedures or systems coincided with the predefined study-period boundaries. The LIS used for data extraction was Bizmed 4 (Sinerji Bilişim, Istanbul, Turkey, version 4.23.11.0009). Data were extracted between 2 and 6 March 2026 by a medical biochemistry specialist who was also a study investigator. The extracted data included the month and year of specimen rejection, specimen type, recorded time of rejection, requesting clinical unit, recorded rejection reason, and the corresponding rejection and total specimen counts. All data meeting the predefined eligibility criteria within the study period were included, and no additional subsampling was performed. The study was conducted in accordance with the Declaration of Helsinki and was approved by the Istanbul Atlas University Non-Interventional Clinical Research Ethics Committee (decision no. 02/14, dated 23 February 2026). The study is reported in accordance with the Strengthening the Reporting of Observational Studies in Epidemiology (STROBE) statement and the REporting of studies Conducted using Observational Routinely collected health Data (RECORD) extension [11,12].

### 2.2. Data Classification

The primary outcome was specimen rejection recorded in the routine LIS during pre-analytical specimen assessment. For the present analysis, data extraction was restricted to spot and 24 h urine specimens, serum specimens, and whole-blood or plasma specimens collected in EDTA-, citrate-, or heparin-containing tubes. Other specimen types, including stool, sputum, smear specimens, body fluids, and other non-selected specimen categories, were not included in the LIS query and were therefore not extracted. Following application of these specimen-type eligibility criteria, both rejection counts and the corresponding eligible specimen counts were stratified by study period, requesting clinical unit, and time interval. For denominator assignment, specimen counts were first categorized according to the specimen type recorded in the LIS as 24 h urine, spot urine, EDTA whole blood, citrate whole blood, heparin whole blood, EDTA plasma, citrate plasma, heparin plasma, or serum. These categories were then consolidated into four analytical specimen groups: urine, whole blood, plasma, and serum. Rejected specimens were additionally classified by rejection reason; for analyses involving rejection reason, the corresponding denominator specimen counts were defined according to the relevant specimen types, as described in Section 2.3. No patient-level diagnostic codes, disease phenotyping algorithms, or record linkage procedures were used.

Unit of analysis: The unit of observation and of analysis throughout this study is the specimen, not the patient. Because the laboratory information system extract consisted of aggregated counts, specimens could not be linked to the individuals from whom they were taken, and no patient identifier was available at any stage. Two consequences follow and are stated here rather than left implicit. First, a single patient may contribute several specimens to the same stratum, particularly among inpatients and intensive care patients, in whom repeated sampling is routine, so the specimens counted within a stratum are not statistically independent. Second, no denominator in this study represents a number of individuals, and the rejection rates reported below are therefore rates per eligible specimen and must not be read as a proportion of patients affected. The implications of the first point for the regression models are addressed in Section 2.5 and again among the limitations.

Study Period: The study period was divided into three phases. The entirety of 2018 and 2019 was classified as the pre-pandemic period; the interval from 1 January 2020 to 30 June 2021 as the pandemic period; and the interval from 1 July 2021 to the end of 2023 as the normalization period. January 2020 was selected as the beginning of the pandemic period to account for the transitional interval between the first reported cases of COVID-19 in China in late 2019 and the introduction of official pandemic-related restrictions in Türkiye in March 2020. 1 July 2021 was defined as the beginning of the normalization period in line with the officially announced nationwide transition to normalization and the lifting of major pandemic-related restrictions in Türkiye.

Clinical unit: The clinical unit from which the specimen request originated was classified into four groups: outpatient clinics, inpatient wards, the emergency department, and the intensive care units.

Time interval: The recorded time of specimen rejection was classified into three intervals: 00:00–07:59 (T1), 08:00–15:59 (T2), and 16:00–23:59 (T3).

Rejection reason: Rejection reasons were identified from the detailed rejection codes recorded in the LIS. These codes were based on the Turkish Ministry of Health Laboratory Errors Classification System. For analysis, the recorded rejection reasons were grouped into five principal categories: hemolysis, clotting, insufficient specimen volume, incorrect collection tube, and other reasons. Hemolysis, clotting, insufficient specimen volume, and incorrect collection tube corresponded to codes L17, L18, L16, and L10, respectively; all other rejection codes represented in the eligible dataset were grouped under “other reasons”. The “other reasons” category comprised specimens rejected due to recollection, inappropriately collected specimen, an empty specimen container, inappropriate transport conditions, transport time exceeding the defined maximum allowable interval, incorrect patient identification, incorrect barcoding, collection from the wrong patient, lipemia, icterus, and specimens that were not delivered to the laboratory. Hemolysis-related specimen rejection was based on visual inspection of centrifuged specimens before analysis. Specimens with visually apparent hemolysis were rejected, whereas specimens that underwent analysis and were subsequently assigned a hemolysis index by the analyzer were reported with an appropriate hemolysis comment rather than recorded as rejected specimens.

### 2.3. Calculation of Rejection Rates

Rejection rates were calculated both overall for each period and within period-specific subgroups defined by clinical unit, time interval, rejection reason, both individually and in combinations.

For analyses not incorporating rejection reason, the rejection rate was calculated as: Rejection rate (%) = (Number of rejected specimens/Number of corresponding eligible specimens) × 100.

For analyses incorporating rejection reason, prespecified reason-specific denominators were used according to specimen type. Four specimen-type groups were considered: urine, whole blood, serum, and plasma. Reason-specific rejection rates were calculated as follows: Hemolysis rejection rate (%) = [Number of hemolyzed specimens/(Number of serum specimens + Number of plasma specimens)] × 100. Clotting rejection rate (%) = [Number of clotted specimens/(Number of whole-blood specimens + Number of plasma specimens)] × 100. Rejection rate for insufficient specimen volume, incorrect collection tube, and other reasons (%) = (Number of specimens rejected for the respective reason/Total number of eligible specimens) × 100.

These calculations were applied within the corresponding study period, clinical unit, time interval, or combined analytical subgroup, as appropriate.

### 2.4. Reporting Framework, Data Provenance, and Quality-Indicator Definitions

Where applicable, the rejection indicators and their interpretation were considered in relation to the ISO 15189:2022 quality-management framework and the IFCC Working Group on Laboratory Errors and Patient Safety (WG-LEPS) Model of Quality Indicators [13,14]. The LIS output consisted of routinely recorded laboratory data provided as aggregated counts stratified by the variables available for each extract, including year and month of rejection, specimen type, requesting clinical unit, recorded rejection time, and rejection reason, rather than individual specimen or patient-level records. Accordingly, no patient-level clinical variables, diagnostic codes or linkage to external databases were used in the study.

The original LIS variables, including detailed rejection codes, were subsequently recategorized into the predefined analytical groupings for study period, clinical unit, time interval, and rejection reason, as described in Section 2.2. The rejection-code structure used in the LIS was implemented in 2017 and remained unchanged throughout the study period (2018–2023). The rejection-code set had been reviewed and approved by the institutional quality commission before routine implementation. Staff were trained in the use of these codes at six-month intervals and additionally when needed. No separate quantitative validation study or performance measures for the rejection-code system were available.

Because the study relied on routinely recorded LIS data, occasional incomplete or inaccurate source data entries cannot be completely excluded. Information not captured in predefined LIS fields was not available for retrospective analysis.

Before analysis, the extracted dataset was reviewed in full for internal consistency and the plausibility of the recorded variables. No dedicated duplicate-record analysis or formal automated range- or logic-check procedure was performed. No inconsistent or impossible entries requiring correction or exclusion were identified during the data review. The six consecutive time intervals available in the original LIS output were combined pairwise to generate the three predefined analytical time intervals (T1, T2, and T3) described in Section 2.2. Other recategorization procedures were performed according to the predefined analytical groupings described in Section 2.2. Because specimen types outside the eligibility criteria were not selected during the LIS query, they were not included in the extracted dataset; therefore, the number of specimens outside the predefined eligibility criteria was not available.

Efforts to address potential sources of bias. Several features of the design were adopted specifically to limit bias, and they are set out together so that the reader can weigh them against the limitations reported in Section 5. Selection of specimens was governed by a single set of specimen-type eligibility criteria applied uniformly at the level of the database query and identically across the three study periods, so that no period was screened more or less stringently than another. All eligible records within the study period were included, with no subsampling, so no selection was exercised by the investigators after extraction. Because the three periods differ in both duration and specimen volume, every cross-period comparison was based on rates with period-specific denominators rather than on absolute counts, which removes the most obvious source of confounding by volume. Differential misclassification across periods was addressed at the level of the data source rather than statistically: the rejection-code structure was implemented in 2017, before the study window opened, and remained unchanged throughout, and the laboratory retained the same supervising specialist, the same tube suppliers, the same transport arrangements and the same analytical platforms across the six years, so that a change in coding or handling practice coinciding with a period boundary is unlikely. What these measures cannot address is confounding by variables absent from the extract, notably staffing, workload and patient case-mix; this residual confounding is unquantifiable from aggregate data and is stated as such in Section 5.

### 2.5. Statistical Analysis

Statistical analyses were performed using IBM SPSS Statistics for Windows, version 27.0 (IBM Corp., Armonk, NY, USA). Rejection counts, specimen counts, and rejection rates were presented descriptively for each study period according to clinical unit, time interval, and rejection reason. For statistical analysis, low-frequency individual rejection reasons were combined into the “other reasons” category to avoid sparse categories in the comparative models. This composite category included lipemia and icterus, which were retained because they were recorded as specimen-rejection reasons in the routine LIS; their inclusion was not intended to imply a pre-analytical handling etiology. Because the three study periods differed in duration and specimen volume, absolute rejection counts were not used for direct cross-period comparisons; instead, comparisons were based on rejection rates calculated using the corresponding eligible specimen denominators defined in Section 2.3. For each analysis level, observed rejection rates were first used to identify the two categories with the highest crude rates. Adjusted analyses were then performed using negative-binomial regression models with a log link and the logarithm of the corresponding eligible specimen denominator as an offset. Main effects of study period, clinical unit, time interval, and rejection reason were evaluated in the relevant models. Interaction analyses were subsequently performed for clinical unit × time interval, clinical unit × rejection reason, and time interval × rejection reason, as well as for the three-way interaction among clinical unit, time interval, and rejection reason. Pairwise comparisons were interpreted only when the corresponding model-level main effect or interaction was statistically significant. Model-based estimated marginal means were used to identify the categories with the highest and second-highest adjusted rejection rates, and pairwise comparisons were performed between these two categories. The resulting estimates were exponentiated to obtain adjusted rejection-rate ratios (RRs) with 95% confidence intervals (CIs). Pairwise *p*-values were Bonferroni-adjusted. A *p*-value <0.05 was considered statistically significant. Given the large number of specimens included in the analysis, statistical significance was interpreted together with the magnitude and precision of the adjusted RRs and their 95% CIs, rather than based on *p*-values alone. As the study included all eligible routinely collected LIS data available for the predefined study period rather than a sampled subset, no formal sample-size calculation was performed. Because patient-level covariates were not available in the aggregated LIS dataset, regression-model adjustment was limited to study period, clinical unit, time interval, and rejection reason, as applicable to each analysis.

Model assumptions and sensitivity analyses: The negative-binomial specification was chosen in preference to Poisson regression because the rejection counts were overdispersed relative to the Poisson mean-variance identity, a feature expected here both from heterogeneity between strata and from the repeated sampling of individual patients described in Section 2.2; the dispersion parameter was estimated from the data in every model. The models nevertheless treat the counts within a stratum as independent, since the aggregated extract contained no patient identifier by which clustering could be accommodated. Where several specimens originate from the same patient, this assumption is not strictly satisfied, and the effect is to make the reported confidence intervals narrower than they would be under a specification allowing for within-patient correlation; the point estimates themselves are not biased by clustering. The comparisons emphasized in the Discussion involve rate ratios well away from unity, and the direction of this effect is therefore known even though its magnitude cannot be estimated from these data. No sensitivity analyses were performed. In particular, the calendar boundaries defining the three study periods were not varied, the transitional months around each boundary were not excluded, and no alternative count specification was fitted; the consequences of this are considered in Section 5.

### 2.6. Data Inclusion, Exclusions, and Missing Data

Data inclusion was based on the specimen-type eligibility criteria described in Section 2.2, which were applied at the time of the LIS query. Specimen types outside these criteria were therefore not extracted, and their total number was not available. Because the LIS output consisted of aggregated counts rather than individual specimen-level records, the total number of individual records extracted or excluded could not be determined, and no dedicated record-level duplicate analysis was performed. No missing or unclassifiable values were identified for requesting clinical unit, time interval, rejection reason, or denominator/specimen-type information in the extracted data. No imputation was required.

## 3. Results

### 3.1. Overall Rejection Rates Across Study Periods

**Figure 1:**
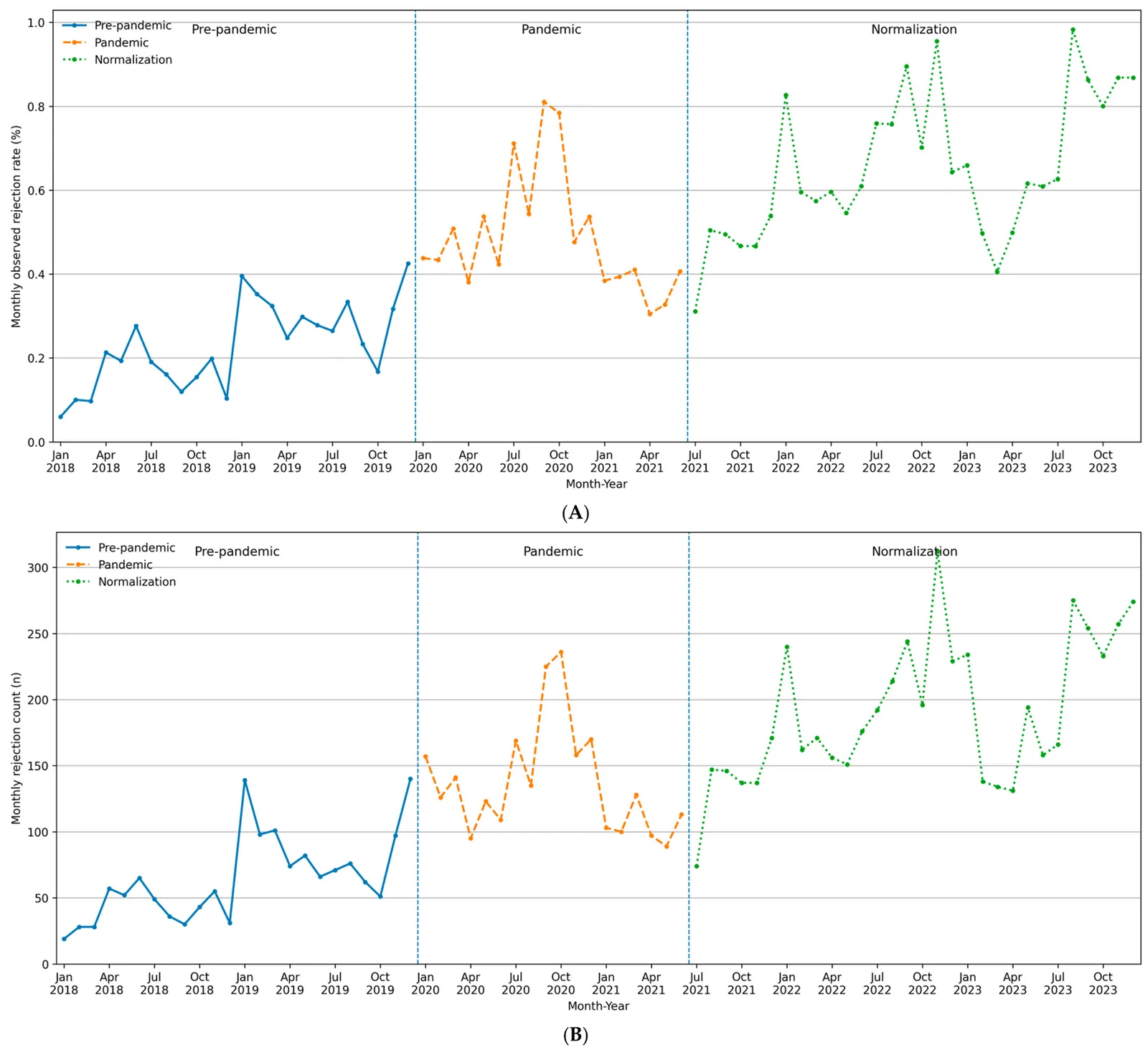
(**A**) Monthly observed specimen rejection rates across the six-year study period; (**B**) Monthly specimen rejection counts across the six-year study period.

The overall rejection rate increased from 0.23% in the pre-pandemic to 0.49% during the pandemic and 0.65% in the normalization period (Figure 1A). Monthly rejection counts were also generally higher during the pandemic and normalization periods than in the pre-pandemic period, although substantial month-to-month variability was observed (Figure 1B). In the adjusted pairwise comparison, the rejection rate was higher during the pandemic period than in the pre-pandemic period (RR = 2.20, 95% CI: 1.96–2.46, *p* < 0.001). The normalization period showed a further increase compared with the pandemic period (RR = 1.42, 95% CI: 1.27–1.59, *p* < 0.001) (Table 1).

**Figure 2:**
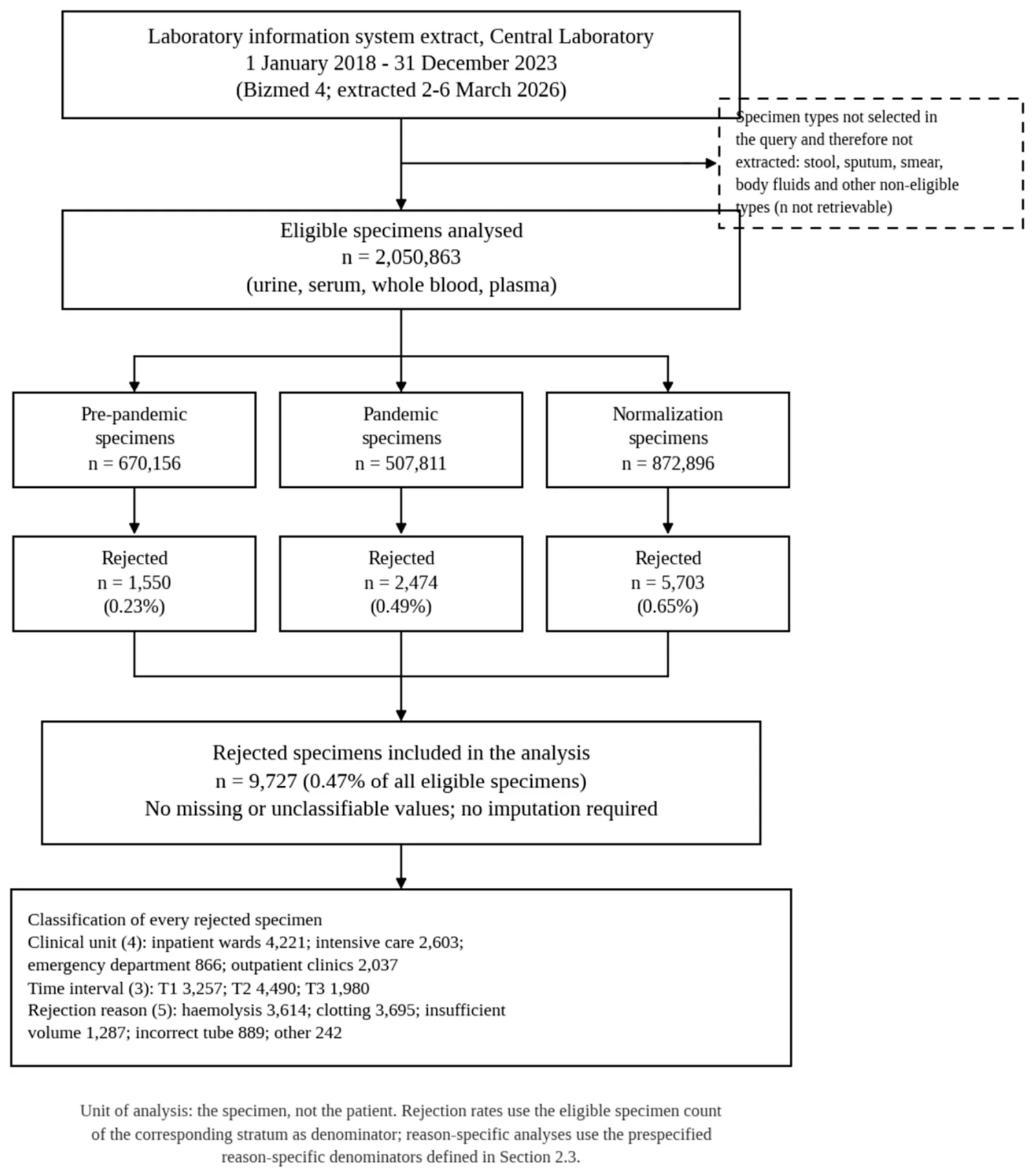
Flow of specimens from the laboratory information system extract to the analyzed dataset, 1 January 2018 to 31 December 2023. Specimen types outside the eligibility criteria were not selected by the database query and were therefore not extracted, so their number is not retrievable. The unit of analysis is the specimen; counts do not represent numbers of patients.

The flow of specimens from the database extract to the analyzed dataset is shown in Figure 2. The extract covered the six-year study period in full. After the specimen-type eligibility criteria had been applied at the level of the query, 2,050,863 eligible specimens remained: 670,156 in the pre-pandemic period, 507,811 during the pandemic and 872,896 during the normalization period. Of these, 9727 were rejected, giving an overall rejection rate of 0.47% across the six years, distributed as 1550 rejections in the pre-pandemic period, 2474 during the pandemic and 5703 during normalization. Every rejected specimen was classifiable by clinical unit, time interval and rejection reason; no record was lost at this stage, no value was missing or unclassifiable, and no imputation was required. Specimen types falling outside the eligibility criteria were not selected by the query and were therefore never extracted, so the number excluded on that ground cannot be reported, as indicated in Figure 2 and discussed in Section 5.

### 3.2. Rejection Rates by Clinical Unit

Across the six-year study period, the emergency department had the highest observed total rejection rate among clinical units (1.10%), followed by intensive care units (0.90%) (Table 2). After adjustment for study period, time interval, and rejection reason, clinical unit remained significantly associated with rejection rates (*p* < 0.001), and the highest adjusted RR was observed for intensive care units (Table 1).

When each study period was evaluated separately, clinical-unit effect in adjusted models remained significantly associated with rejection rates in all three periods (all *p* < 0.001). In contrast to the crude period-specific findings, the highest adjusted rejection rate was observed in the emergency department only in the pre-pandemic period, whereas intensive care units had the highest adjusted rejection rates during the pandemic and normalization periods. Pairwise comparisons between the emergency department and intensive care units showed that the difference was statistically significant only during the pandemic period, with intensive care units showing a higher rejection rate than the emergency department (RR = 1.34, 95% CI: 1.00–1.80, *p* = 0.047). No significant difference was observed between these two units in the pre-pandemic period (emergency department vs. intensive care units: RR = 1.42, 95% CI: 0.96–2.11, *p* = 0.105) or in the normalization period (intensive care units vs. emergency department: RR = 1.15, 95% CI: 0.92–1.42, *p* = 0.587) (Table 1).

### 3.3. Rejection Rates by Time Interval

Across the six-year study period, the highest total rejection rate by time interval was found in T1 (00:00–07:59), followed by T3 (16:00–23:59) and T2 (08:00–15:59). After adjustment time interval remained significantly associated with rejection rates (*p* < 0.001). In contrast to the observed total rates, T1 showed a lower adjusted RR than T2, whereas T3 did not differ significantly from T2 (Table 1).

In the period-specific adjusted analyses, time interval was significantly associated with rejection rates in the pre-pandemic and pandemic periods. In both the pre-pandemic and pandemic periods, the highest adjusted rejection rate was observed in T2, followed by T3; however, the difference between these two intervals was not statistically significant (*p* = 0.485 and *p* = 0.094, respectively). In the normalization period, the model-level effect of time interval was not statistically significant; therefore, pairwise comparisons were not further interpreted (Table 1).

### 3.4. Rejection Rates by Sample Rejection Reason

Across the six-year study period, hemolysis had the highest observed total rejection rate among rejection reasons, followed by clotting. Rejection reason remained significantly associated with rejection rates in the adjusted analysis (*p* < 0.001). In the adjusted pairwise comparison, hemolysis showed a higher rejection rate than clotting, which had the second-highest adjusted rejection rate (RR = 1.48, 95% CI: 1.29–1.69, *p* < 0.001) (Table 1).

The period-specific adjusted analyses showed a significant effect of rejection reason in all three periods (all *p* < 0.001). Although the observed rates were highest for clotting in the pre-pandemic and pandemic periods, hemolysis had the highest adjusted rejection rate in all three periods. Hemolysis did not differ significantly from clotting in either the pre-pandemic period (RR = 1.18, 95% CI: 0.88–1.60, *p* = 1.000) or the pandemic period (RR = 1.14, 95% CI: 0.90–1.46, *p* = 1.000). In the normalization period, however, hemolysis showed a significantly higher adjusted rejection rate than clotting (RR = 1.97, 95% CI: 1.65–2.35, *p* < 0.001) (Table 1).

### 3.5. Rejection Rates by Clinical Unit × Time Interval Combination

In the pre-pandemic period, the highest observed rejection rate among clinical unit–time interval combinations was seen in the emergency department during T2 (0.77%), followed by the emergency department during T3 (0.54%). However, the clinical unit × time interval interaction was not statistically significant in the adjusted model (*p* = 0.095); therefore, pairwise comparisons between unit–time combinations were not further interpreted (Table 1 and Table 3).

A different pattern emerged during the pandemic period, in which intensive care units–T2 became the leading observed unit–time combination, followed by emergency department–T2 (Table 3). After adjustment, intensive care units–T2 remained the leading combination, followed by intensive care units–T3. The adjusted rejection rate in intensive care units–T2 was significantly higher than that in intensive care units–T3 (RR = 1.84, 95% CI: 1.06–3.19, *p* = 0.014), indicating that the increase in rejection rate within intensive care units was most pronounced during T2 (Table 1). Although emergency department–T2 was also among the highest observed combinations, T2 did not represent a clearly distinct high-rejection interval within the emergency department, as pairwise comparisons between time intervals in the emergency department were not statistically significant (all *p* > 0.05).

In the normalization period, the highest observed rejection rate was seen in emergency department–T2, followed by intensive care units–T2 (Table 3). Although the clinical unit × time interval interaction was significant in the adjusted model (*p* = 0.044), adjusted pairwise comparisons did not identify a clearly dominant unit–time combination. Intensive care units–T2 ranked highest after adjustment, but it was not significantly different from the next leading combinations, including intensive care units–T3 and emergency department–T2 (*p* = 1.000 for both) (Table 1).

### 3.6. Rejection Rates by Clinical Unit × Sample Rejection Reason Combinations

In the pre-pandemic period, emergency department–hemolysis had the highest observed rejection rate among clinical unit–rejection reason combinations, followed by intensive care units–clotting (Table 4). This pattern was retained after adjustment. Emergency department–hemolysis showed a higher adjusted rejection rate than intensive care units–clotting (RR = 2.51, 95% CI: 1.12–5.61, *p* = 0.006) (Table 1). Within the emergency department, hemolysis was also higher than clotting (RR = 3.11, 95% CI: 1.33–7.29, *p* < 0.001), and among hemolysis-related rejections, the emergency department showed a higher rate than inpatient wards (RR = 2.53, 95% CI: 1.23–5.18, *p* < 0.001).

During the pandemic period, emergency department–hemolysis had the highest observed and adjusted rejection rate among clinical unit–rejection reason combinations, followed by intensive care units–clotting in the observed data. However, the adjusted difference between these two leading combinations was not statistically significant (RR = 1.43, 95% CI: 0.85–2.42, *p* = 1.000) (Table 1 and Table 4). Additional targeted comparisons showed that emergency department–hemolysis was not significantly separated either within the emergency department, when compared with emergency department–clotting (RR = 1.92, 95% CI: 0.87–4.22, *p* = 0.481), or within hemolysis-related rejections, when compared with intensive care units–hemolysis (RR = 1.45, 95% CI: 0.82–2.55, *p* = 1.000).

In the normalization period, emergency department–hemolysis had the highest observed rejection rate among clinical unit–rejection reason combinations, followed by inpatient wards–hemolysis (Table 4). The adjusted estimates again identified emergency department–hemolysis as the leading combination, followed by intensive care units–hemolysis and inpatient wards–hemolysis. Emergency department–hemolysis showed a significantly higher adjusted rejection rate than both intensive care units–hemolysis (RR = 1.89, 95% CI: 1.19–3.00, *p* < 0.001) and inpatient wards–hemolysis (RR = 1.90, 95% CI: 1.34–2.71, *p* < 0.001) (Table 1). Within the emergency department, hemolysis was also significantly higher than clotting (RR = 4.52, 95% CI: 2.60–7.85, *p* < 0.001).

For clotting-related rejections, the highest adjusted rejection rate was observed in intensive care units in all three periods. However, this unit-specific pattern was not equally distinct across periods. In the pre-pandemic and pandemic periods, intensive care units–clotting was not clearly separated from all other high-rate clinical units, whereas in the normalization period, intensive care units showed a more distinct clotting-related pattern, with higher adjusted rejection rates than the other clinical units (Table 5).

### 3.7. Rejection Rates by Time Interval × Sample Rejection Reason Combinations

The time interval × rejection reason interaction was significant in all three study periods (all *p* < 0.001), indicating that time-related patterns differed according to the specific rejection reason. Because hemolysis and clotting represented the dominant rejection reasons across the analyses, comparisons were focused on these two categories.

For hemolysis-related rejections, the highest adjusted rejection rate was observed in T2 during the pre-pandemic and normalization periods, whereas T3 ranked highest during the pandemic period. However, in each period, the leading hemolysis-related interval was not significantly different from the second-ranked interval, indicating that hemolysis did not show a clearly dominant time interval after adjustment (Table 1).

For clotting-related rejections, T2 had the highest adjusted rejection rate in the pre-pandemic and pandemic periods, but it was not significantly different from the second-ranked interval in either period. In the normalization period, however, T1 became the leading interval for clotting-related rejections and was significantly higher than other intervals (Table 1).

### 3.8. Rejection Rates by Clinical Unit × Time Interval × Sample Rejection Reason Combinations

In the observed three-way combinations, emergency department–T2–hemolysis had the highest rejection rate in the pre-pandemic period, followed by emergency department–T3–hemolysis. During the pandemic period, the leading observed combination shifted to intensive care units–T2–clotting, followed by emergency department–T3–hemolysis. In the normalization period, emergency department–T2–hemolysis again had the highest observed rejection rate, followed by emergency department–T3–hemolysis (Appendix A Table A1, Table A2 and Table A3).

The clinical unit × time interval × rejection reason interaction was significant in the pre-pandemic period (*p* < 0.001), but not during the pandemic (*p* = 0.191) or normalization period (*p* = 0.051). Accordingly, period-specific three-way patterns were not consistently observed across the study periods, and detailed adjusted pairwise comparisons were not further interpreted for the pandemic and normalization periods.

## 4. Discussion

This retrospective analysis of routinely collected LIS data showed a progressive increase in pre-analytical specimen rejection rates across the predefined pre-pandemic, pandemic, and normalization periods. Given the very large specimen volume, interpretation focused on adjusted rejection-rate ratios and 95% CIs rather than *p*-values alone. The adjusted rejection rate was 2.20-fold higher during the pandemic than in the pre-pandemic period (95% CI: 1.96–2.46) and increased a further 1.42-fold in the normalization period compared with the pandemic period (95% CI: 1.27–1.59). Patterns differed by clinical unit, time interval, and rejection reason. Because the study was observational and lacked patient- and staff-level covariates, these temporal associations should not be interpreted as demonstrating that the pandemic caused the observed changes. Pandemic-related disruption and longer-term institutional or operational changes remain plausible, non-mutually exclusive explanations for the observed temporal pattern.

A distinguishing feature of this analysis is that it does not rely on a single aggregate rejection rate but instead examines study period, clinical unit, time interval, and rejection reason both individually and in two-way and three-way combinations. Much of the existing literature on pre-analytical quality has reported overall hemolysis or aggregate specimen rejection rates in isolation [15,16], which, while informative, cannot reveal how error patterns are redistributed across a health care system under changing conditions. By examining these dimensions at multiple levels, the present study demonstrates that pre-analytical error patterns are not static but vary according to clinical setting, time of day, rejection reason, and study period. Notably, whereas the two-way analyses yielded more consistent and interpretable adjusted patterns, the three-way clinical unit × time interval × rejection reason interaction was significant only in the pre-pandemic period; consistent adjusted three-way patterns were therefore not obtained across periods, and interpretation was deliberately confined to the two-way level. These heterogeneous patterns identify where when, and which types of pre-analytical specimen rejection were concentrated across the study periods, while the mechanisms underlying these differences could not be directly evaluated from the available data.

The overall rejection rate increased nearly threefold from the pre-pandemic to the normalization period, and the monthly observed rejection rates (Figure 1A) supported this progressive increase across periods. Monthly rejection counts (Figure 1B) likewise showed generally higher absolute numbers of rejected specimens during the later study periods, although both measures exhibited month-to-month variability. The monthly trends allowed visual inspection of temporal variability, but seasonality was not formally assessed. Because the three study periods differed in duration, absolute rejection counts were not treated as comparable measures across periods, and the cross-period interpretation rests on rejection rates with period-specific denominators and on adjusted rate ratios. Although the observed rejection rate in the normalization period remained below 1%, consistent with the range commonly reported in general laboratory quality surveys [1,2], the magnitude and persistence of this increase are noteworthy. The fact that the highest overall rejection rate was observed in the normalization period suggests that the overall trend cannot be attributed solely to disruptions occurring during the acute phase of the pandemic. A plausible explanation, consistent with the institutional context of this study, is that longer-term changes including the hospital’s structural expansion, the addition of new clinical departments, and an increasingly complex and heterogeneous patient population may have contributed to the continued increase, while the pandemic introduced an additional disruption to pre-analytical processes. This interpretation is consistent with the well-established understanding that a substantial proportion of laboratory errors originate in the pre-analytical phase, which is particularly sensitive to changes in staffing, patient acuity, and workflow [17,18,19].

The emergency department consistently showed the highest observed rejection rate in all three study periods, a pattern that is well documented in the pre-analytical literature and is generally attributed to high patient turnover, time pressure, frequent blood collection through intravenous catheters rather than direct venipuncture, and comparatively limited opportunities for real-time supervision of sampling technique [20,21]. After adjustment, however, this unit-level ranking was not stable: the emergency department retained the highest adjusted rejection rate only in the pre-pandemic period, whereas intensive care units ranked highest during the pandemic and normalization periods, with the difference between these two units reaching statistical significance only during the pandemic (RR = 1.34, 95% CI: 1.00–1.80). This divergence between observed and adjusted rankings indicates that part of the apparent emergency department excess reflected the distribution of time intervals and rejection reasons across units rather than a uniformly higher unit-level risk, and that these two high-acuity settings shared a similarly elevated pre-analytical risk during the pandemic and normalization periods, despite differences in their rejection-reason patterns.

In contrast, when specimens were considered by absolute count rather than by rate, inpatient wards consistently accounted for the highest number of rejected specimens in every study period. This divergence between the unit with the highest rejection rate and the unit with the highest rejection count is not contradictory but reflects two distinct and complementary dimensions of pre-analytical quality: the rejection rate, normalized to the total number of relevant specimens, more directly reflects the proportional risk of rejection and the quality of the sampling process after accounting for specimen volume, whereas the raw rejection count reflects the absolute operational burden placed on the laboratory and is influenced by the number of specimens processed. We would argue that the rate-based metric is the more appropriate indicator of underlying process quality for cross-period and cross-unit comparisons, because it relates the number of rejections to the volume of relevant specimens; in the present study this caution is reinforced by the unequal durations of the three periods, which preclude any direct cross-period comparison of raw counts. The count-based metric nevertheless remains operationally important for resource allocation and for identifying where the greatest absolute burden of recollection, delayed testing, and additional laboratory work is generated. Reporting both metrics together, as done here, therefore provides a more complete picture than either metric alone.

At the opposite end of the unit-level spectrum, outpatient clinics maintained the lowest overall rejection rate among the clinical units in all three study periods. This finding is consistent with guidance emphasizing standardized and closely supervised phlebotomy practices as important components of pre-analytical quality [22,23]. In our institution, the outpatient phlebotomy unit is located adjacent to the laboratory specimen-reception area, allowing short transport times, regular supervision, and rapid feedback when potential pre-analytical problems are identified. Such direct oversight is more difficult to maintain in inpatient wards and other clinical settings, where blood collection is performed across multiple locations. The persistence of the lowest overall rejection rate in outpatient clinics throughout all three study periods suggests that proximity to the laboratory and more controlled workflow may have contributed to better overall pre-analytical performance.

A more nuanced picture emerged for time intervals. In the observed data, the overnight and early-morning interval (00:00–07:59) showed the highest overall rejection rate in all three study periods, whereas the daytime interval (08:00–15:59) accounted for the highest rejection count, consistent with the substantially greater specimen volume processed during standard working hours. After adjustment, however, T1 did not remain the dominant interval: the highest adjusted rates in the pre-pandemic and pandemic periods were observed in T2, without significant separation from the second-ranked interval, and in the normalization period the main effect of time interval was not statistically significant. The time-interval effect was therefore context-dependent and became informative when evaluated together with clinical unit or rejection reason. The clearest unit-specific time pattern was observed during the pandemic, when intensive care unit rejections peaked during T2, with a significantly higher adjusted rejection rate than during T3 (RR = 1.84, 95% CI: 1.06–3.19). This pattern is compatible with pandemic-related daytime workload intensity, high specimen volume, and pressure on collection and tube-mixing procedures within this unit, rather than with a generalized overnight vulnerability. Fatigue-, staffing-, and circadian-related explanations for the elevated observed overnight rates, although supported by the broader occupational literature on shift work [24], are not directly corroborated by the adjusted analyses and should therefore be regarded as secondary hypotheses to be interpreted with caution.

In the observed data, clot-related rejections had the highest rate and count in the pre-pandemic and pandemic periods, with a shift toward hemolysis in the normalization period. The adjusted analyses refined this picture: hemolysis ranked highest among rejection reasons in all three study periods, but it clearly separated from clotting only in the normalization period (RR = 1.97, 95% CI: 1.65–2.35), whereas the adjusted rates for the two reasons were statistically indistinguishable in the pre-pandemic and pandemic periods. The adjusted predominance of hemolysis is more consistent with reports from many clinical chemistry laboratories, in which hemolysis is frequently identified as the most common reason for specimen rejection [16,25], and suggests that the observed prominence of clotting in the earlier periods was period-specific and partly denominator-dependent rather than a stable institutional pattern across the entire study interval.

Several factors may have contributed to the prominence of clot-related rejections, particularly during the pandemic period. It should be emphasized, however, that the outcome analyzed here is an in vitro specimen rejection event, and clot formation in an anticoagulated collection tube is generally influenced strongly by collection and mixing procedures rather than by patient physiology alone. Infection-control pressures and efforts to minimize close-contact time during phlebotomy may have increased the likelihood of hurried venipuncture, prolonged or difficult draws, and inadequate mixing of blood with anticoagulant, all of which promote clot formation in collection tubes. Although SARS-CoV-2 infection is associated with endothelial injury and a hypercoagulable state [26,27], the aggregated data analyzed here contain no patient-level information on infection status, coagulation parameters, or anticoagulant treatment; any contribution of patient-related coagulability to in vitro clot formation therefore remains a speculative hypothesis that cannot be tested in this dataset, and the increase in clotted specimens during the pandemic should not be attributed to SARS-CoV-2–related hypercoagulability. Accordingly, the observed pandemic-period increase in clot-related specimen rejections should be interpreted as an association at the aggregate level rather than evidence of a COVID-19-specific biological effect; collection- and technique-related mechanisms represent the more plausible primary explanation for this pattern.

Beyond these biological and technique-related explanations, the pandemic also represented a broader psychological burden for health care staff that may have affected pre-analytical performance. Health care workers directly involved in the care of patients with suspected or confirmed COVID-19 have been reported to experience elevated levels of distress, anxiety, depression, and insomnia during this period [7]. The cognitive and emotional burden associated with these conditions, superimposed on an already high clinical workload, may have made it more difficult to maintain the sustained attention and procedural consistency required for pre-analytical tasks such as venipuncture and tube handling. Under this interpretation, the increases in hemolysis- and clot-related rejection rates during the pandemic period may have partly reflected challenges in maintaining procedural vigilance among staff working under sustained psychological strain.

The subsequent shift toward hemolysis as the leading rejection reason in the normalization period is less readily explained by pandemic-specific mechanisms and may instead reflect a greater contribution of workload- and technique-related factors. In vitro hemolysis is strongly influenced by pre-analytical practices, and traumatic or technically suboptimal venipuncture is a recognized preventable contributor [5,6,17]. The clearest clinical expression of this normalization period hemolysis pattern was centered on the emergency department: emergency department–hemolysis was the leading clinical unit–rejection reason combination in both the observed and adjusted analyses, with significantly higher adjusted rates than intensive care unit–hemolysis (RR = 1.89, 95% CI: 1.19–3.00) and inpatient ward–hemolysis (RR = 1.90, 95% CI: 1.34–2.71); within the emergency department, the adjusted rate for hemolysis also exceeded that for clotting more than fourfold (RR = 4.52, 95% CI: 2.60–7.85). Notably, hemolysis-related rejections did not show a single dominant time interval in any period after adjustment, suggesting a problem distributed across the day rather than confined to particular shifts. In the context of expanding clinical activity, sustained pressure on emergency department blood-collection processes—characterized by high patient turnover, catheter-based collection, and time pressure [20,21]—therefore emerges as the most concrete normalization period target for quality monitoring and preventive intervention.

A complementary unit-specific pattern concerned clot-related rejections, which were consistently centered on intensive care units: the highest adjusted clotting-related rejection rate was observed in intensive care units in all three study periods. This ICU-centered pattern was not, however, equally distinct across periods. In the pre-pandemic and pandemic periods, intensive care units were not clearly separated from the other high-rate clinical units, whereas in the normalization period the separation became more distinct, with intensive care units showing a significantly higher adjusted clotting-related rate than inpatient wards (RR = 1.53, 95% CI: 1.10–2.14). The time-interval structure of clotting also changed over the study interval: T2 ranked highest for clotting-related rejections in the pre-pandemic and pandemic periods without significant separation, whereas clotting became more T1-centered in the normalization period, with the overnight and early-morning interval significantly exceeding the other intervals (RR = 1.41 versus T3, 95% CI: 1.02–1.96). Rather than a single stable rejection pattern, intensive care units were thus characterized by the persistent coexistence of clotting- and hemolysis-related rejections whose relative prominence varied across time intervals and study periods. During the pandemic, the prominence of the intensive care units–T2 combination may have reflected daytime workload intensity together with collection-related factors, including specimens being set aside or transferred before adequate mixing with anticoagulant [28].

Although outpatient clinics maintained the lowest overall rejection rate throughout the study, supplementary observed analyses suggested that clotting was the most frequent rejection reason in this setting during the pre-pandemic and pandemic periods, shifting toward hemolysis in the normalization period. Because these observations derive from crude stratum-specific rates in a low-rejection setting and were not a focus of the adjusted analyses, they should be regarded as a secondary observation. They may indicate a localized vulnerability in the collection or mixing of anticoagulated specimens rather than a stable unit-specific feature, and the subsequent shift toward hemolysis suggests that the predominant rejection pattern in outpatient clinics changed over time.

Taken together, these findings indicate that overall rejection rates alone are insufficient to fully characterize pre-analytical rejection patterns, and that time interval in particular should not be interpreted in isolation: its effect was context-dependent and became meaningful mainly when evaluated together with clinical unit or rejection reason. From a quality-management perspective, this supports complementing routine aggregate monitoring with clinical unit– and rejection reason–specific time-interval surveillance capable of detecting emerging changes in rejection rates and reasons. In the present data, emergency department–hemolysis during the normalization period and intensive care unit–centered clotting represent the most concrete such monitoring targets. Real-time workload indicators, flexible allocation of phlebotomy support during periods of increased demand, and continuously updated quality dashboards may facilitate earlier operational responses. In this sense, the pandemic functioned as a system-level stress test, underscoring that laboratory quality is closely linked to wider clinical workflows and that quality-management structures should be sufficiently resilient and adaptable to respond to future health system disruptions.

These findings also have implications for internationally harmonized laboratory quality management. ISO 15189:2022 places risk-based management, control of nonconformities, evaluation of performance, and continual improvement within the quality and competence framework for medical laboratories, while the IFCC WG-LEPS quality-indicator model promotes standardized data collection and benchmarking of extra-analytical errors [13,14]. Accordingly, the rejection-rate denominators used in this study were defined according to the population of specimens at risk for the corresponding rejection mechanism rather than by using a single undifferentiated denominator for every indicator. This approach improves interpretability across rejection categories; however, exact comparability with external laboratories still depends on harmonized local definitions, LIS coding practices, and denominator availability. Future multicenter studies should therefore prospectively map local LIS rejection codes to standardized IFCC quality-indicator definitions before benchmarking across institutions.

## 5. Limitations

This study has several limitations. First, its retrospective, single-center observational design limits causal inference and external generalizability. Second, the three study periods differed in duration; consequently, raw rejection counts are not directly comparable across periods, although rate-based analyses used period-specific denominators. Third, analyses were based on aggregated routinely collected LIS data rather than patient- or staff-level observations, precluding adjustment for patient case-mix, staffing, workload, collection technique, and other potential confounders. Fourth, routine LIS data are vulnerable to incomplete recording, miscoding, misclassification, and changes in coding practice over time. The extent and direction of any resulting information bias cannot be quantified from the available aggregate data. A related consequence is that the number of specimens falling outside the eligibility criteria is not recoverable, because ineligible specimen types were never selected by the database query; the flow diagram in Figure 2 therefore begins at the eligible pool rather than at the point of retrieval. The LIS rejection-code system had been reviewed and approved by the institutional quality commission before routine implementation; however, no separate quantitative validation study or performance measures for the coding system were available.

Several variables that could plausibly influence pre-analytical outcomes, including phlebotomist experience and training, staff turnover and real-time staffing density, venipuncture technique, catheter use and tube mixing were not directly captured. Patient-level information such as confirmed COVID-19 status, coagulation parameters, anticoagulant treatment, and venous access difficulty was also unavailable; for this reason, patient-related coagulability could not be evaluated as a cause of clot-related rejections, and such mechanisms were treated as untestable hypotheses. Similarly, the psychological burden experienced by health care staff was not directly assessed. In addition, changes in hospital size, clinical activity, patient case-mix, and workload across the study periods were not quantitatively measured. Although monthly rejection rates were plotted to describe temporal variation, formal time-series modeling or seasonal decomposition was not performed; temporal patterns were therefore interpreted descriptively. Two further methodological limitations follow from the aggregated nature of the extract and are stated explicitly because their direction is known. The unit of analysis is the specimen and no patient identifier was available, so specimens originating from the same individual could not be identified and the regression models treat counts within a stratum as independent. Since repeated sampling of the same patient is routine in inpatient wards and intensive care units, this assumption is not strictly satisfied, and the effect is to render the reported confidence intervals narrower than they would be under a specification allowing for within-patient correlation; the point estimates are unaffected, and the comparisons emphasized here involve rate ratios sufficiently far from unity that their direction is not in question, but the stated precision should be regarded as an upper bound. In addition, no sensitivity analysis was undertaken: the calendar boundaries of the three study periods were not varied, the transitional months surrounding each boundary were not excluded, and no alternative count specification was fitted, so the robustness of the period contrasts to these choices has not been demonstrated. Finally, the calendar-based definition of the study periods may not fully reflect the gradual nature of epidemiological, behavioral, and organizational transitions. Accordingly, the observed temporal and unit-specific differences are well supported by the data, whereas the biological, technical, and organizational explanations proposed for these differences should be interpreted as hypotheses rather than confirmed causal mechanisms. Despite these limitations, the study has several important strengths, including its large overall sample size, six-year observation period, and multidimensional analytic approach with effect-size–based interpretation, which enabled the identification of patterns across clinical units, time intervals, rejection reasons, and study periods that would not have been apparent from overall rejection rates alone. In addition, because the study relied on routinely collected LIS data, independent source-document validation of every rejection event was not feasible. The analysis was therefore dependent on the accuracy and consistency of routine rejection coding, which should be considered when interpreting category-specific estimates.

## 6. Conclusions

Pre-analytical specimen rejection increased progressively from the pre-pandemic period to the pandemic and normalization periods, with the highest overall rejection rate observed in the normalization period; in adjusted terms, the rejection rate more than doubled during the pandemic and increased by a further 42% thereafter. The pattern of this increase differed across clinical units, time intervals, and rejection reasons. The emergency department had the highest observed rejection rate in all three study periods, whereas intensive care units ranked highest after adjustment during the pandemic and normalization periods, and inpatient wards accounted for the highest rejection count. Time interval did not emerge as a consistently dominant factor after adjustment; its effect was context-dependent and became meaningful mainly in combination with clinical unit or rejection reason, as illustrated by the daytime peak of intensive care unit rejections during the pandemic. Hemolysis and clotting were the predominant rejection reasons: hemolysis ranked highest after adjustment in all periods and separated significantly from clotting in the normalization period, during which emergency department–hemolysis emerged as the leading clinical unit–rejection reason combination, while clotting remained centered on intensive care units and became more concentrated in the overnight and early-morning interval.

The persistence and further increase in specimen rejection during the normalization period indicate that the observed deterioration was not confined to the acute pandemic period and may also reflect longer-term institutional and operational changes. These findings support the view that preparedness for future large-scale health system crises similar to COVID-19 should extend beyond maintaining laboratory analytical capacity and should also include detailed monitoring of pre-analytical processes. Rather than assigning blanket priority to particular clinical units or hours of the day, quality monitoring should be stratified by clinical unit, time interval, and rejection reason in combination, with emergency department–hemolysis during the normalization period and intensive care unit–related clotting representing the most concrete current targets. Because rejection rates reflect the frequency of specimen rejection, whereas rejection counts indicate the total burden of recollection, testing delays, and additional workload—and because counts are not directly comparable across periods of unequal duration—evaluating both measures together, with rates as the primary basis for cross-period comparison, may provide a more comprehensive assessment of pre-analytical quality. Given that rejection patterns may continue to change after a health system crisis, incorporating this stratified, effect-size–based approach into routine quality monitoring following major health system disruptions may help identify emerging pre-analytical problems earlier and more accurately, guide preventive interventions more effectively, and contribute to the development of more resilient and adaptable quality-management systems.

## Figures and Tables

**Table 1:** Comparison of observed and adjusted leading rejection-rate categories.

			Observed Rejection Rate Ranking		Adjusted Rejection Rate Ranking				
	Effect/Interaction	Effect *p*-Value	Highest Category (A)	Second-Highest Category (B)	Highest Category (C)	Second-Highest Category (D)	Adjusted RR	95% CI	Pairwise *p*-Value
**6 years**	Study period	<0.001	Normalization	Pandemic	Normalization	Pandemic	1.42	1.27–1.59	<0.001
	Clinical unit	<0.001	ED	ICU	ICU	ED	1.08	0.91–1.27	1.000
	Time interval	<0.001	T1	T3	T2	T3	1.05	0.94–1.18	0.809
	Rejection reason	<0.001	Hemolysis	Clotting	Hemolysis	Clotting	1.48	1.29–1.69	<0.001
**Pre-pandemic**	Clinical unit	<0.001	ED	ICU	ED	ICU	1.42	0.96–2.11	0.105
	Time interval	0.046	T1	T3	T2	T3	1.15	0.90–1.47	0.485
	Rejection reason	<0.001	Clotting	Hemolysis	Hemolysis	Clotting	1.18	0.88–1.60	1.000
	Clinical unit × Time interval	0.095	ED-T2	ED-T3	Not interpreted	Not interpreted	–	–	–
	Clinical unit × Rejection reason	<0.001	ED- Hemolysis	ICU- Clotting	ED- Hemolysis	ICU- Clotting	2.51	1.12–5.61	0.006
	Clinical unit × Rejection reason (Hemolysis)	<0.001	ED	IPW	ED	IPW	2.53	1.23–5.18	<0.001
	Clinical unit × Rejection reason (Clotting)	<0.001	ICU	ED	ICU	IPW	1.14	0.60–2.15	1.000
	Time interval × Rejection reason	<0.001	T1- Clotting	T3- Hemolysis	T2- Hemolysis	T3- Hemolysis	1.12	0.57–2.20	1.000
	Time interval × Rejection reason (Hemolysis)	<0.001	T3	T2	T2	T3	1.12	0.57–2.20	1.000
	Time interval × Rejection reason (Clotting)	<0.001	T1	T3	T2	T1	1.01	0.60–1.71	1.000
**Pandemic**	Clinical unit	<0.001	ED	ICU	ICU	ED	1.34	1.00–1.80	0.047
	Time interval	<0.001	T1	T3	T2	T3	1.21	0.98–1.50	0.094
	Rejection reason	<0.001	Clotting	Hemolysis	Hemolysis	Clotting	1.14	0.90–1.46	1.000
	Clinical unit × Time interval	0.009	ICU-T2	ED-T2	ICU-T2	ICU-T3	1.84	1.06–3.19	0.014
	Clinical unit × Rejection reason	<0.001	ED- Hemolysis	ICU- Clotting	ED- Hemolysis	ICU- Clotting	1.43	0.85–2.42	1.000
	Clinical unit × Rejection reason (Hemolysis)	<0.001	ED	ICU	ED	ICU	1.45	0.82–2.55	1.000
	Clinical unit × Rejection reason (Clotting)	<0.001	ICU	ED	ICU	ED	1.34	0.65–2.78	1.000
	Time interval × Rejection reason	<0.001	T1- Clotting	T1- Hemolysis	T3- Hemolysis	T2- Hemolysis	1.01	0.62–1.65	1.000
	Time interval × Rejection reason (Hemolysis)	<0.001	T1	T3	T3	T2	1.01	0.62–1.65	1.000
	Time interval × Rejection reason (Clotting)	<0.001	T1	T3	T2	T3	1.24	0.74–2.09	1.000
**Normalization**	Clinical unit	<0.001	ED	ICU	ICU	ED	1.15	0.92–1.42	0.587
	Time interval	0.112	T1	T3	Not interpreted	Not interpreted	–	–	–
	Rejection reason	<0.001	Hemolysis	Clotting	Hemolysis	Clotting	1.97	1.65–2.35	<0.001
	Clinical unit × Time interval	0.044	ED-T2	ICU-T2	ICU-T2	ICU-T3	1.06	0.68–1.65	1.000
	Clinical unit × Rejection reason	<0.001	ED- Hemolysis	IPW- Hemolysis	ED- Hemolysis	ICU- Hemolysis	1.89	1.19–3.00	<0.001
	Clinical unit × Rejection reason (Hemolysis)	<0.001	ED	IPW	ED	ICU	1.89	1.19–3.00	<0.001
	Clinical unit × Rejection reason (Clotting)	<0.001	ICU	IPW	ICU	IPW	1.53	1.10–2.14	0.001
	Time interval × Rejection reason	<0.001	T3- Hemolysis	T1- Clotting	T2- Hemolysis	T3- Hemolysis	1.02	0.72–1.46	1.000
	Time interval × Rejection reason (Hemolysis)	<0.001	T3	T1	T2	T3	1.02	0.72–1.46	1.000
	Time interval × Rejection reason (Clotting)	<0.001	T1	T3	T1	T3	1.41	1.02–1.96	0.021

Observed categories were ranked according to crude rejection-rate percentages. Adjusted RR indicates the model-based rejection-rate ratio of the adjusted highest category (C) compared with the adjusted second-highest category (D) in each row, as estimated from the corresponding adjusted negative-binomial regression model. Effect *p*-value refers to the significance of the corresponding main effect or interaction term in the adjusted negative-binomial regression model. Pairwise comparisons were interpreted only when the effect *p*-value was <0.05. Pairwise *p*-value refers to the Bonferroni-adjusted comparison between categories C and D. An RR > 1 indicates a higher rejection rate in category C than in category D, whereas an RR < 1 indicates a lower rejection rate in category C than in category D. RR, rejection-rate ratio; CI, confidence interval; ED, emergency department; ICU, intensive care units; IPW, inpatient wards. Time intervals were defined as T1 (00:00–07:59), T2 (08:00–15:59), and T3 (16:00–23:59).

**Table 2:** Specimen rejection counts and observed rejection rates across the six-year study period.

Variable	Category	Rejection Count (n)	Specimen Count (n)	Total Rejection Rate (%)
Study period	Pre-pandemic	1550	670,156	0.23
	Pandemic	2474	507,811	0.49
	Normalization	5703	872,896	0.65
Clinical unit	Inpatient wards	4221	577,919	0.73
	Intensive care units	2603	288,218	0.90
	Emergency department	866	78,618	1.10
	Outpatient clinics	2037	1,106,108	0.18
Time interval	T1 (00:00–07:59)	3257	451,227	0.72
	T2 (08:00–15:59)	4490	1,226,034	0.37
	T3 (16:00–23:59)	1980	373,602	0.53
Rejection reason	Hemolysis	3614	972,764	0.37
	Clotting	3695	1,116,828	0.33
	Insufficient specimen volume	1287	2,050,863	0.06
	Incorrect collection tube	889	2,050,863	0.04
	Other reasons	242	2,050,863	0.01

Total Rejection Rate (%): The proportion of rejected specimens to the total number of specimens in the relevant category across the entire study period, expressed as a percentage.

**Table 3:** Specimen rejection counts and observed rates by clinical unit and time interval, stratified by study period.

Study Period	Clinical Unit		Time Intervals			TOTAL
			T1 (00:00–07:59)	T2 (08:00–15:59)	T3 (16:00–23:59)	
**Pre-pandemic**	**Inpatient** **wards**	Rejection count (n)	217	379	135	731
		Specimen count (n)	75,834	87,593	36,202	199,629
		Rejection Rate (%)	0.29	0.43	0.37	0.37
	**Intensive** **care units**	Rejection count (n)	218	76	65	359
		Specimen count (n)	55,279	23,184	18,879	97,342
		Rejection Rate (%)	0.39	0.33	0.34	0.37
	**Emergency** **department**	Rejection count (n)	14	74	47	135
		Specimen count (n)	4621	9607	8742	22,970
		Rejection Rate (%)	0.30	0.77	0.54	0.59
	**Outpatient** **clinics**	Rejection count (n)	18	263	44	325
		Specimen count (n)	13,711	273,787	62,717	350,215
		Rejection Rate (%)	0.13	0.10	0.07	0.09
	**TOTAL**	Rejection count (n)	467	792	291	1550
		Specimen count (n)	149,445	394,171	126,540	670,156
		Rejection Rate (%)	0.31	0.20	0.23	0.23
**Pandemic**	**Inpatient** **wards**	Rejection count (n)	327	540	142	1009
		Specimen count (n)	59,260	72,870	23,146	155,276
		Rejection Rate (%)	0.55	0.74	0.61	0.65
	**Intensive** **care units**	Rejection count (n)	325	326	112	763
		Specimen count (n)	45,786	19,175	12,740	77,701
		Rejection Rate (%)	0.71	1.70	0.88	0.98
	**Emergency** **department**	Rejection count (n)	37	84	78	199
		Specimen count (n)	4502	7621	7511	19,634
		Rejection Rate (%)	0.82	1.10	1.04	1.01
	**Outpatient** **clinics**	Rejection count (n)	18	380	105	503
		Specimen count (n)	10,151	201,814	43,235	255,200
		Rejection Rate (%)	0.18	0.19	0.24	0.20
	**TOTAL**	Rejection count (n)	707	1330	437	2474
		Specimen count (n)	119,699	301,480	86,632	507,811
		Rejection Rate (%)	0.59	0.44	0.50	0.49
**Normalization**	**Inpatient** **wards**	Rejection count (n)	939	1012	530	2481
		Specimen count (n)	87,627	90,185	45,202	223,014
		Rejection Rate (%)	1.07	1.12	1.17	1.11
	**Intensive** **care units**	Rejection count (n)	989	272	220	1481
		Specimen count (n)	70,195	23,867	19,113	113,175
		Rejection Rate (%)	1.41	1.14	1.15	1.31
	**Emergency** **department**	Rejection count (n)	90	234	208	532
		Specimen count (n)	7936	13,956	14,122	36,014
		Rejection Rate (%)	1.13	1.68	1.47	1.48
	**Outpatient** **clinics**	Rejection count (n)	65	850	294	1209
		Specimen count (n)	16,325	402,375	81,993	500,693
		Rejection Rate (%)	0.40	0.21	0.36	0.24
	**TOTAL**	Rejection count (n)	2083	2368	1252	5703
		Specimen count (n)	182,083	530,383	160,430	872,896
		Rejection Rate (%)	1.14	0.45	0.78	0.65

**Table 4:** Specimen rejection counts and observed rates by clinical unit and rejection reason, stratified by study period.

Study Period	Clinical Unit		Sample Rejection Reasons					
			Hemolysis	Clotting	Insufficient Specimen Volume	Incorrect Collection Tube	Other Reasons	TOTAL
**Pre-pandemic**	**Inpatient** **wards**	Rejection count (n)	264	307	86	51	23	731
		Specimen count (n)	88,092	126,210	199,629	199,629	199,629	199,629
		Relevant specimen count (n)	64974a, 23118b	23118b, 103092c	64974a, 23118b, 103092c, 8445d	64974a, 23118b, 103092c, 8445d	64974a, 23118b, 103092c, 8445d	64974a, 23118b, 103092c, 8445d
		Rejection Rate (%)	0.30	0.24	0.04	0.03	0.01	0.37
	**Intensive** **care units**	Rejection count (n)	76	203	30	36	14	359
		Specimen count (n)	37,820	66,902	97,342	97,342	97,342	97,342
		Relevant specimen count (n)	22476a, 15344b	15344b, 51558c	22476a, 15344b, 51558c, 7964d	22476a, 15344b, 51558c, 7964d	22476a, 15344b, 51558c, 7964d	22476a, 15344b, 51558c, 7964d
		Rejection Rate (%)	0.20	0.30	0.03	0.04	0.01	0.37
	**Emergency** **department**	Rejection count (n)	86	31	9	4	5	135
		Specimen count (n)	10,480	12,569	22,970	22,970	22,970	22,970
		Relevant specimen count (n)	9058a, 1422b	1422b, 11147c	9058a, 1422b, 11147c, 1343d	9058a, 1422b, 11147c, 1343d	9058a, 1422b, 11147c, 1343d	9058a, 1422b, 11147c, 1343d
		Rejection Rate (%)	0.82	0.25	0.04	0.02	0.02	0.59
	**Outpatient** **clinics**	Rejection count (n)	76	147	62	35	5	325
		Specimen count (n)	169,837	161,357	350,215	350,215	350,215	350,215
		Relevant specimen count (n)	143774a, 26063b	26063b, 135294c	143774a, 26063b, 135294c, 45084d	143774a, 26063b, 135294c, 45084d	143774a, 26063b, 135294c, 45084d	143774a, 26063b, 135294c, 45084d
		Rejection Rate (%)	0.04	0.09	0.02	0.01	0.00	0.09
	**TOTAL**	Rejection count (n)	502	688	187	126	47	1550
		Specimen count (n)	306,229	367,038	670,156	670,156	670,156	670,156
		Relevant specimen count (n)	240282a, 65947b	65947b, 301091c	240282a, 65947b, 301091c, 62836d	240282a, 65947b, 301091c, 62836d	240282a, 65947b, 301091c, 62836d	240282a, 65947b, 301091c, 62836d
		Rejection Rate (%)	0.16	0.19	0.03	0.02	0.01	0.23
**Pandemic**	**Inpatient** **wards**	Rejection count (n)	382	384	108	114	21	1009
		Specimen count (n)	73,522	99,262	155,276	155,276	155,276	155,276
		Relevant specimen count (n)	49858a, 23664b	23664b, 75598c	49858a, 23664b, 75598c, 6156d	49858a, 23664b, 75598c, 6156d	49858a, 23664b, 75598c, 6156d	49858a, 23664b, 75598c, 6156d
		Rejection Rate (%)	0.52	0.39	0.07	0.07	0.01	0.65
	**Intensive** **care units**	Rejection count (n)	212	350	99	83	19	763
		Specimen count (n)	35,605	49,744	77,701	77,701	77,701	77,701
		Relevant specimen count (n)	21707a, 13898b	13898b, 35846c	21707a, 3898b, 35846c, 6250d	21707a, 13898b, 35846c, 6250d	21707a, 13898b, 35846c, 6250d	21707a, 13898b, 35846c, 6250d
		Rejection Rate (%)	0.60	0.70	0.13	0.11	0.02	0.98
	**Emergency** **department**	Rejection count (n)	118	64	7	8	2	199
		Specimen count (n)	10,389	10,723	19,634	19,634	19,634	19,634
		Relevant specimen count (n)	8041a, 2348b	2348b, 8375c	8041a, 2348b, 8375c, 870d	8041a, 2348b, 8375c, 870d	8041a, 2348b, 8375c, 870d	8041a, 2348b, 8375c, 870d
		Rejection Rate (%)	1.14	0.60	0.04	0.04	0.01	1.01
	**Outpatient** **clinics**	Rejection count (n)	98	183	170	35	17	503
		Specimen count (n)	132,783	115,154	255,200	255,200	255,200	255,200
		Relevant specimen count (n)	112433a, 20350b	20350b, 94804c	112433a, 20350b, 94804c, 27613d	112433a, 20350b, 94804c, 27613d	112433a, 20350b, 94804c, 27613d	112433a, 20350b, 94804c, 27613d
		Rejection Rate (%)	0.07	0.16	0.07	0.01	0.01	0.20
	**TOTAL**	Rejection count (n)	810	981	384	240	59	2474
		Specimen count (n)	252,299	274,883	507,811	507,811	507,811	507,811
		Relevant specimen count (n)	192039a, 60260b	60260b, 214623c	192039a, 60260b, 214623c, 40889d	192039a, 60260b, 214623c, 40889d	192039a, 60260b, 214623c, 40889d	192039a, 60260b, 214623c, 40889d
		Rejection Rate (%)	0.32	0.36	0.08	0.05	0.01	0.49
**Normalization**	**Inpatient** **wards**	Rejection count (n)	1015	897	260	275	34	2481
		Specimen count (n)	91,776	151,445	223,014	223,014	223,014	223,014
		Relevant specimen count (n)	62765a, 29011b	29011b, 122434c	62765a, 29011b, 122434c, 8804d	62765a, 29011b, 122434c, 8804d	62765a, 29011b, 122434c, 8804d	62765a, 29011b, 122434c, 8804d
		Rejection Rate (%)	1.11	0.59	0.12	0.12	0.02	1.11
	**Intensive** **care units**	Rejection count (n)	415	765	139	125	37	1481
		Specimen count (n)	50,163	73,633	113,175	113,175	113,175	113,175
		Relevant specimen count (n)	29977a, 20186b	20186b, 53447c	29977a, 20186b, 53447c, 9565d	29977a, 20186b, 53447c, 9565d	29977a, 20186b, 53447c, 9565d	29977a, 20186b, 53447c, 9565d
		Rejection Rate (%)	0.83	1.04	0.12	0.11	0.03	1.31
	**Emergency** **department**	Rejection count (n)	389	89	24	23	7	532
		Specimen count (n)	18,995	19,858	36,014	36,014	36,014	36,014
		Relevant specimen count (n)	14623a, 4372b	4372b, 15486c	14623a, 4372b, 15486c, 1533d	14623a, 4372b, 15486c, 1533d	14623a, 4372b, 15486c, 1533d	14623a, 4372b, 15486c, 1533d
		Rejection Rate (%)	2.05	0.45	0.07	0.06	0.02	1.48
	**Outpatient** **clinics**	Rejection count (n)	483	275	293	100	58	1209
		Specimen count (n)	253,302	229,971	500,693	500,693	500,693	500,693
		Relevant specimen count (n)	216298a, 37004b	37004b, 192967c	216298a, 37004b, 192967c, 54424d	216298a, 37004b, 192967c, 54424d	216298a, 37004b, 192967c, 54424d	216298a, 37004b, 192967c, 54424d
		Rejection Rate (%)	0.19	0.12	0.06	0.02	0.01	0.24
	**TOTAL**	Rejection count (n)	2302	2026	716	523	136	5703
		Specimen count (n)	414,236	474,907	872,896	872,896	872,896	872,896
		Relevant specimen count (n)	323663a, 90573b	90573b, 384334c	323663a, 90573b, 384334c, 74326d	323663a, 90573b, 384334c, 74326d	323663a, 90573b, 384334c, 74326d	323663a, 90573b, 384334c, 74326d
		Rejection Rate (%)	0.56	0.43	0.08	0.06	0.02	0.65

Relevant specimen count (n): Specimen types associated with observed rejection reasons. a: Serum, b: Plasma, c: Whole Blood Sample, d: Urine.

**Table 5:** Specimen rejection counts and observed rates by time interval and rejection reason, stratified by study period.

Study Period	Time Intervals		Sample Rejection Reasons					
			Hemolysis	Clotting	Insufficient Specimen Volume	Incorrect Collection Tube	Other Reasons	TOTAL
**Pre-pandemic**	**T1** **(00:00–07:59)**	Rejection count (n)	100	271	29	43	24	467
		Specimen count (n)	70,420	92,287	149,445	149,445	149,445	149,445
		Relevant specimen count (n)	51793a, 18627b	18627b, 73660c	51793a, 18627b, 73660c, 5365d	51793a, 18627b, 73660c, 5365d	51793a, 18627b, 73660c, 5365d	51793a, 18627b, 73660c, 5365d
		Rejection Rate (%)	0.14	0.29	0.02	0.03	0.02	0.31
	**T2** **(08:00–15:59)**	Rejection count (n)	299	301	127	51	14	792
		Specimen count (n)	185,347	204,140	394,171	394,171	394,171	394,171
		Relevant specimen count (n)	146367a, 38980b	38980b, 165160c	146367a, 38980b, 165160c, 43664d	146367a, 38980b, 165160c, 43664d	146367a, 38980b, 165160c, 43664d	146367a, 38980b, 165160c, 43664d
		Rejection Rate (%)	0.16	0.15	0.03	0.01	0.00	0.20
	**T3** **(16:00–23:59)**	Rejection count (n)	103	116	31	32	9	291
		Specimen count (n)	50,462	70,611	126,540	126,540	126,540	126,540
		Relevant specimen count (n)	42122a, 8340b	8340b, 62271c	42122a, 8340b, 62271c, 13807d	42122a, 8340b, 62271c, 13807d	42122a, 8340b, 62271c, 13807d	42122a, 8340b, 62271c, 13807d
		Rejection Rate (%)	0.20	0.16	0.02	0.03	0.01	0.23
	**TOTAL**	Rejection count (n)	502	688	187	126	47	1550
		Specimen count (n)	306,229	367,038	670,156	670,156	670,156	670,156
		Relevant specimen count (n)	240282a, 65947b	65947b, 301091c	240282a, 65947b, 301091c, 62836d	240282a, 65947b, 301091c, 62836d	240282a, 65947b, 301091c, 62836d	240282a, 65947b, 301091c, 62836d
		Rejection Rate (%)	0.16	0.19	0.03	0.02	0.01	0.23
**Pandemic**	**T1** **(00:00–07:59)**	Rejection count (n)	252	349	36	53	17	707
		Specimen count (n)	61,221	73,885	119,699	119,699	119,699	119,699
		Relevant specimen count (n)	42603a, 18618b	18618b, 55267c	42603a, 18618b, 55267c, 3211d	42603a, 18618b, 55267c, 3211d	42603a, 18618b, 55267c, 3211d	42603a, 18618b, 55267c, 3211d
		Rejection Rate (%)	0.41	0.47	0.03	0.04	0.01	0.59
	**T2** **(08:00–15:59)**	Rejection count (n)	406	473	271	150	30	1330
		Specimen count (n)	152,726	153,563	301,480	301,480	301,480	301,480
		Relevant specimen count (n)	118548a, 34178b	34178b, 119385c	118548a, 34178b, 119385c, 29369d	118548a, 34178b, 119385c, 29369d	118548a, 34178b, 119385c, 29369d	118548a, 34178b, 119385c, 29369d
		Rejection Rate (%)	0.27	0.31	0.09	0.05	0.01	0.44
	**T3** **(16:00–23:59)**	Rejection count (n)	152	159	77	37	12	437
		Specimen count (n)	38,352	47,435	86,632	86,632	86,632	86,632
		Relevant specimen count (n)	30888a, 7464b	7464b, 39971c	30888a, 7464b, 39971c, 8309d	30888a, 7464b, 39971c, 8309d	30888a, 7464b, 39971c, 8309d	30888a, 7464b, 39971c, 8309d
		Rejection Rate (%)	0.40	0.34	0.09	0.04	0.01	0.50
	**TOTAL**	Rejection count (n)	810	981	384	240	59	2474
		Specimen count (n)	252,299	274,883	507,811	507,811	507,811	507,811
		Relevant specimen count (n)	192039a, 60260b	60260b, 214623c	192039a, 60260b, 214623c, 40889d	192039a, 60260b, 214623c, 40889d	192039a, 60260b, 214623c, 40889d	192039a, 60260b, 214623c, 40889d
		Rejection Rate (%)	0.32	0.36	0.08	0.05	0.01	0.49
**Normalization**	**T1** **(00:00–07:59)**	Rejection count (n)	629	1109	163	139	43	2083
		Specimen count (n)	87,371	117,114	182,083	182,083	182,083	182,083
		Relevant specimen count (n)	59651a, 27720b	27720b, 89394c	59651a, 27720b, 89394c, 5318d	59651a, 27720b, 89394c, 5318d	59651a, 27720b, 89394c, 5318d	59651a, 27720b, 89394c, 5318d
		Rejection Rate (%)	0.72	0.95	0.09	0.08	0.02	1.14
	**T2** **(08:00–15:59)**	Rejection count (n)	1147	523	358	265	75	2368
		Specimen count (n)	258,504	267,543	530,383	530,383	530,383	530,383
		Relevant specimen count (n)	208880a, 49624b	49624b, 217919c	208880a, 49624b, 217919c, 53960d	208880a, 49624b, 217919c, 53960d	208880a, 49624b, 217919c, 53960d	208880a, 49624b, 217919c, 53960d
		Rejection Rate (%)	0.44	0.20	0.07	0.05	0.01	0.45
	**T3** **16:00–23:59)**	Rejection count (n)	526	394	195	119	18	1252
		Specimen count (n)	55,132	90,250	160,430	160,430	160,430	160,430
		Relevant specimen count (n)	55132a, 13229b	13229b, 77021c	55132a, 13229b, 77021c, 15048d	55132a, 13229b, 77021c, 15048d	55132a, 13229b, 77021c, 15048d	55132a, 13229b, 77021c, 15048d
		Rejection Rate (%)	0.95	0.44	0.12	0.07	0.01	0.78
	**TOTAL**	Rejection count (n)	2302	2026	716	523	136	5703
		Specimen count (n)	414,236	474,907	872,896	872,896	872,896	872,896
		Relevant specimen count (n)	323663a, 90573b	90573b, 384334c	323663a, 90573b, 384334c, 74326d	323663a, 90573b, 384334c, 74326d	323663a, 90573b, 384334c, 74326d	323663a, 90573b, 384334c, 74326d
		Rejection Rate (%)	0.56	0.43	0.08	0.06	0.02	0.65

Relevant specimen count (n): Specimen types associated with observed rejection reasons. a: Serum, b: Plasma, c: Whole Blood Sample, d: Urine.

## Data Availability

The underlying routinely collected LIS data are not publicly available because they are subject to ethical, privacy, and institutional restrictions. Requests for access may be directed to the corresponding author and will require approval under applicable institutional and ethics procedures.

## Appendix A

**Table A1:** Specimen rejection counts and observed rates by clinical unit, time interval and rejection reason, in pre-pandemic period.

Pre-Pandemic Period			Sample Rejection Reasons				
Clinical Unit	Time Intervals		Hemolysis	Clotting	Insufficient Specimen Volume	Incorrect Collection Tube	Other Reasons
**Inpatient** **wards**	T1 (00:00–07:59)	Rejection count (n)	40	134	13	20	10
		Specimen count (n)	34,197	45,859	75,834	75,834	75,834
		Rejection Rate (%)	0.117	0.292	0.017	0.026	0.013
	T2 (08.00–15.59)	Rejection count (n)	179	113	59	18	10
		Specimen count (n)	41,064	55,658	87,593	87,593	87,593
		Rejection Rate (%)	0.436	0.203	0.067	0.021	0.011
	T3 (16.00–23.59)	Rejection count (n)	45	60	14	13	3
		Specimen count (n)	12,831	24,693	36,202	36,202	36,202
		Rejection Rate (%)	0.351	0.243	0.039	0.036	0.008
**Intensive** **care units**	T1 (00:00–07:59)	Rejection count (n)	39	131	14	21	13
		Specimen count (n)	27,744	37,605	55,279	55,279	55,279
		Rejection Rate (%)	0.141	0.348	0.025	0.038	0.024
	T2 (08.00–15.59)	Rejection count (n)	19	40	12	4	1
		Specimen count (n)	5716	15,837	23,184	23,184	23,184
		Rejection Rate (%)	0.332	0.253	0.052	0.017	0.004
	T3 (16.00–23.59)	Rejection count (n)	18	32	4	11	0
		Specimen count (n)	4360	13,460	18,879	18,879	18,879
		Rejection Rate (%)	0.413	0.238	0.021	0.058	0.000
**Emergency** **department**	T1 (00:00–07:59)	Rejection count (n)	9	4	1	0	0
		Specimen count (n)	2133	2525	4621	4621	4621
		Rejection Rate (%)	0.422	0.158	0.022	0.000	0.000
	T2 (08.00–15.59)	Rejection count (n)	50	18	3	3	0
		Specimen count (n)	4346	5291	9607	9607	9607
		Rejection Rate (%)	1.150	0.340	0.031	0.031	0.000
	T3 (16.00–23.59)	Rejection count (n)	27	9	5	1	5
		Specimen count (n)	4001	4753	8742	8742	8742
		Rejection Rate (%)	0.675	0.189	0.057	0.011	0.057
**Outpatient** **clinics**	T1 (00:00–07:59)	Rejection count (n)	12	2	1	2	1
		Specimen count (n)	6346	6298	13,711	13,711	13,711
		Rejection Rate (%)	0.189	0.032	0.007	0.015	0.007
	T2 (08.00–15.59)	Rejection count (n)	51	130	53	26	3
		Specimen count (n)	134,221	127,354	273,787	273,787	273,787
		Rejection Rate (%)	0.038	0.102	0.019	0.009	0.001
	T3 (16.00–23.59)	Rejection count (n)	13	15	8	7	1
		Specimen count (n)	29,270	27,705	62,717	62,717	62,717
		Rejection Rate (%)	0.044	0.054	0.013	0.011	0.002

**Table A2:** Specimen rejection counts and observed rates by clinical unit, time interval and rejection reason, in pandemic period.

Pandemic Period			Sample Rejection Reasons				
Clinical Unit	Time Intervals		Hemolysis	Clotting	Insufficient Specimen Volume	Incorrect Collection Tube	Other Reasons
**Inpatient** **wards**	T1 (00:00–07:59)	Rejection count (n)	107	165	25	24	6
		Specimen count (n)	28,249	36,354	59,260	59,260	59,260
		Rejection Rate (%)	0.379	0.454	0.042	0.040	0.010
	T2 (08.00–15.59)	Rejection count (n)	223	163	66	77	11
		Specimen count (n)	36,414	46,732	72,870	72,870	72,870
		Rejection Rate (%)	0.612	0.349	0.091	0.106	0.015
	T3 (16.00–23.59)	Rejection count (n)	52	56	17	13	4
		Specimen count (n)	8859	16,176	23,146	23,146	23,146
		Rejection Rate (%)	0.587	0.346	0.073	0.056	0.017
**Intensive** **care units**	T1 (00:00–07:59)	Rejection count (n)	112	165	9	29	10
		Specimen count (n)	25,679	30,147	45,786	45,786	45,786
		Rejection Rate (%)	0.436	0.547	0.020	0.063	0.022
	T2 (08.00–15.59)	Rejection count (n)	68	147	68	38	5
		Specimen count (n)	6033	11,373	19,175	19,175	19,175
		Rejection Rate (%)	1.127	1.293	0.355	0.198	0.026
	T3 (16.00–23.59)	Rejection count (n)	32	38	22	16	4
		Specimen count (n)	3893	8224	12,740	12,740	12,740
		Rejection Rate (%)	0.822	0.462	0.173	0.126	0.031
**Emergency** **department**	T1 (00:00–07:59)	Rejection count (n)	25	10	1	0	1
		Specimen count (n)	2401	2508	4502	4502	4502
		Rejection Rate (%)	1.041	0.399	0.022	0.000	0.022
	T2 (08.00–15.59)	Rejection count (n)	47	27	4	6	0
		Specimen count (n)	4042	4108	7621	7621	7621
		Rejection Rate (%)	1.163	0.657	0.052	0.079	0.000
	T3 (16.00–23.59)	Rejection count (n)	46	27	2	2	1
		Specimen count (n)	3946	4107	7511	7511	7511
		Rejection Rate (%)	1.166	0.657	0.027	0.027	0.013
**Outpatient** **clinics**	T1 (00:00–07:59)	Rejection count (n)	8	9	1	0	0
		Specimen count (n)	4892	4876	10151	10151	10151
		Rejection Rate (%)	0.164	0.185	0.010	0.000	0.000
	T2 (08.00–15.59)	Rejection count (n)	68	136	133	29	14
		Specimen count (n)	106,237	91,350	201,814	201,814	201,814
		Rejection Rate (%)	0.064	0.149	0.066	0.014	0.007
	T3 (16.00–23.59)	Rejection count (n)	22	38	36	6	3
		Specimen count (n)	21,654	18,928	43,235	43,235	43,235
		Rejection Rate (%)	0.102	0.201	0.083	0.014	0.007

**Table A3:** Specimen rejection counts and observed rates by clinical unit, time interval and rejection reason, in normalization period.

Normalization Period			Sample Rejection Reasons				
Clinical Unit	Time Intervals		Hemolysis	Clotting	Insufficient Specimen Volume	Incorrect Collection Tube	Other Reasons
**Inpatient** **wards**	T1 (00:00–07:59)	Rejection count (n)	273	490	83	71	22
		Specimen count (n)	35,635	59,538	87,627	87,627	87,627
		Rejection Rate (%)	0.766	0.823	0.095	0.081	0.025
	T2 (08.00–15.59)	Rejection count (n)	528	215	116	145	8
		Specimen count (n)	40,535	58,656	90,185	90,185	90,185
		Rejection Rate (%)	1.303	0.367	0.129	0.161	0.009
	T3 (16.00–23.59)	Rejection count (n)	214	192	61	59	4
		Specimen count (n)	15,606	33,251	45,202	45,202	45,202
		Rejection Rate (%)	1.371	0.577	0.135	0.131	0.009
**Intensive** **care units**	T1 (00:00–07:59)	Rejection count (n)	268	581	63	61	16
		Specimen count (n)	39,774	45,462	70,195	70,195	70,195
		Rejection Rate (%)	0.674	1.278	0.090	0.087	0.023
	T2 (08.00–15.59)	Rejection count (n)	92	94	34	37	15
		Specimen count (n)	5779	15,556	23,867	23,867	23,867
		Rejection Rate (%)	1.592	0.604	0.142	0.155	0.063
	T3 (16.00–23.59)	Rejection count (n)	55	90	42	27	6
		Specimen count (n)	4610	12,615	19,113	19,113	19,113
		Rejection Rate (%)	1.193	0.713	0.220	0.141	0.031
**Emergency** **department**	T1 (00:00–07:59)	Rejection count (n)	65	16	3	4	2
		Specimen count (n)	4247	4431	7936	7936	7936
		Rejection Rate (%)	1.530	0.361	0.038	0.050	0.025
	T2 (08.00–15.59)	Rejection count (n)	174	37	11	12	0
		Specimen count (n)	7251	7645	13,956	13,956	13,956
		Rejection Rate (%)	2.400	0.484	0.079	0.086	0.000
	T3 (16.00–23.59)	Rejection count (n)	150	36	10	7	5
		Specimen count (n)	7497	7782	14,122	14,122	14,122
		Rejection Rate (%)	2.001	0.463	0.071	0.050	0.035
**Outpatient** **clinics**	T1 (00:00–07:59)	Rejection count (n)	23	22	14	3	3
		Specimen count (n)	7715	7683	16,325	16,325	16,325
		Rejection Rate (%)	0.298	0.286	0.086	0.018	0.018
	T2 (08.00–15.59)	Rejection count (n)	353	177	197	71	52
		Specimen count (n)	204,939	185,686	402,375	402,375	402,375
		Rejection Rate (%)	0.172	0.095	0.049	0.018	0.013
	T3 (16.00–23.59)	Rejection count (n)	107	76	82	26	3
		Specimen count (n)	40,648	36,602	81,993	81,993	81,993
		Rejection Rate (%)	0.263	0.208	0.100	0.032	0.004
